# Testosterone boosters: a report of a supplement’s misleading labelling claims

**DOI:** 10.17159/2078-516X/2020/v32i1a7426

**Published:** 2020-01-01

**Authors:** RW de Lange

**Affiliations:** Department of Visual Communication, Faculty of Arts and Design, Tshwane University of Technology, Pretoria, South Africa

**Keywords:** deceptive advertising, D-aspartic acid, abuse of scientific information

## Abstract

This paper illustrates how labelling claims of a testosterone booster supplement mislead consumers. The labelling claims misappropriate scientific terminology, exaggerate and misrepresent research as evidence for the product’s purported efficacy.

Nutritional supplements sometimes contain adulterated and undeclared substances. Gabriels et al. reported several incidences where nutritional supplements tested positive for harmful and undeclared substances not indicated on the labels.^[1,2]^ Of 138 nutritional supplements commercially available in South Africa, 47% tested positive for melamine^[1]^ and 54% for fluoxetine.^[2]^ Whilst the median melamine concentration was below the Tolerable Daily Intake (TDI) and the level of fluoxetine contamination low, these substances were not indicated on the labels.^[1]^ Melamine is used in plastics and fertilisers, and artificially boosts the protein level in food.^[3]^ Fluoxetine, a selective serotonin reuptake inhibitor, is used to treat major depression and bulimia nervosa.^[4]^

A Canadian manufacturer of sports supplements promotes a muscle- and testosterone-boosting product in South Africa via retailers and sports and nutritional outlets. The manufacturer’s advertisements use science sounding efficacy claims for this product, supported by images of hypermasculine men. Men suffering from muscle dysmorphia are ideally the intended target audience. This pathological disorder seems to make them more susceptible to appeals for a more muscular physique, and even a brief exposure to the aforementioned imagery, may induce the desired effect.^[5]^ Men with muscle dysmorphia are also more likely to experiment with anabolic steroids and other performance-enhancing substances.^[6]^

The objective of this paper is to illustrate how labelling claims of one such product, a purported testosterone booster, potentially mislead consumers by misusing and exaggerating scientific information. This product, as an illustrative example, is but one of many similar products that misuses the same scientific information that appeared in a reproductive and endocrinology journal.^[7]^ The following sections report on the process, claims and evidence supplied by the advertiser, and conclude by questioning these advertising claims.

## Case report

### The process

An email was sent to the company requesting information on the advertising claims. The information provided did not support their advertising claims. A visit to a store that sold the product, followed by a subsequent email, inquired whether the store holds sufficient evidence for supporting the advertising claims. The store manager received information from the company and via email, presented three scientific articles ^[7, 8, 9]^ and an Evidence and Safety Summary Report as purported evidence for the product’s efficacy claims. The advertiser’s claims were compared against the evidence supplied by the advertiser, and empirical results pertaining to the effect of exogenous D-aspartic acid, the product’s key ingredient, on human testosterone.

The term ‘D-aspartic acid’ (and its derivatives) combined with ‘testosterone’ were used as keywords to search for in vivo studies on exogenous D-aspartic acid and its effect on human testosterone levels. However, the search did not reveal any relevant articles in the Cochrane Database of Systematic Reviews or reputable urology journals to support a notion that exogenous D-aspartic acid will increase testosterone levels in humans. The Aspartates monograph, available from the Natural Medicines Comprehensive Database, does not list D-aspartic acid as a testosterone booster, only stating that people use it for the absorption of supplements and athletic performance. The monograph further cautions that sufficient and reliable information about its safety and effectiveness is not available.^[10]^

### The product’s labelling claims

The labelling claims for the product is about its efficacy, which is shown on a website and the product’s packaging. These claims are that the product can increase, support, stimulate, amplify, and boost testosterone levels. Additional claims are that the product has been clinically researched and that its results are clinically proven.

### The advertiser’s evidence

The first article is a study that deals with rats and not human participants and was therefore dismissed.^[8]^ The second article is a summary of current and previous work on the role of D-aspartic acid in the nervous and endocrine systems of invertebrates and vertebrates, including mammals.^[9]^ However, it does not cover any studies on the advertised testosterone boosting product or any similar products, nor does it look at the increase of testosterone in humans. The third article is a study by Topo et al.^[7]^ and could be considered as the only bona fide attempt at providing evidence for this product’s efficacy claims. It reports on the role and molecular mechanism of D-aspartic acid in the release and synthesis of luteinising hormone (LH), and testosterone in humans and in rats. The fourth document is the manufacturer’s own Evidence and Safety Summary Report which provides summaries of the three previously mentioned studies and lists an additional eight articles. One of these articles is about the nomenclature and symbolism of amino acids and peptides. The other seven articles are about the claimed safety and health benefits of resveratrol, rhodiola, and vitamins. These eight additional articles do not provide any information to support the product’s testosterone boosting claims.

### Are the claims misleading?

The claim regarding D-aspartic acid as a testosterone boosting agent is misleading for several reasons. The advertiser misuses and misrepresents scientific information from the Topo et al.^[7]^ study and contradicts the results of other scientific studies. The advertiser’s claims are interrogated below.

The first argument is that the advertiser misuses scientific information, particularly tabulated data from the Topo et al.^[7]^ study, which are reproduced below.

The total testosterone in the Topo et al.^[7]^ study increased from a mean of 4.5 (± 0.6) ng/ml serum at zero time to 6.4 (± 0.8) ng/ml after 12 days of use. This then decreased to 5.8 (± 0.6) ng/ml after three days of suspension of D-aspartic acid treatment (for the treatment group). The increase from 4.5 to 6.4 translates to an increase of 42%, a value given on the product’s website to validate their claim of increased testosterone levels. The increase from 4.5 ng/ml to 6.4 ng/ml may be statistically significant but will nonetheless not have any noticeable effect on a user. As such, the results may be statistically significant but clinically insignificant. Clinical significance or the practical value of an increase would be when free testosterone levels increase beyond the upper threshold for the consumer’s age group. The Topo et al.^[7]^ study also did not calculate and report on free testosterone, only on total testosterone. Total testosterone consists of testosterone bound to sex hormone binding globulin (SHBG) and free testosterone. Free testosterone is a portion of circulating testosterone that crosses cell membranes, exerts a biologic effect and contributes, inter alia, to muscle strength.

Although there was a statistically significant increase in total testosterone, the increase is still in the lower range of testosterone levels in healthy adult males. It is thus highly unlikely that this small increase, even for men with low levels of testosterone, would produce a discernible effect on their ability to build muscle. What is noteworthy from the Topo et al.^[7]^ study is that the volunteers (treatment = 23; placebo = 20) were tested at an in vitro fertilisation clinic. The mean base levels of total testosterone for the treatment and placebo groups were low, namely 4.6 (±0.5) ng/ml and 4.5 (±0.6) ng/ml. Although the Topo et al.^[7]^ paper states that the test participants were healthy, these base levels are at the lower range of total testosterone for males. The participants did not come from a population of men with total testosterone levels that are spread over the normal range. The normal range of total testosterone levels for men (19–39 years) range from 2.67 ng/ml to 9.29 ng/ml.^[11]^

The second reason for arguing that the claims are misleading is the difference in the time usage of the different products. The participants in the Topo et al.^[7]^ study received a solution with D-aspartic acid for 12 days. The advertiser’s product contained 96 capsules which, according to the package design, equates to a 30 day cycle. As such, the results are not directly transferable.

The third reason for misleading the consumer is that the Topo et al.^[7]^ study used a different product and not the advertiser’s product for testing. The ingredients and the D-aspartic acid dosage (3120 mg/day) are similar, except that the advertiser added resveratrol and rhodiola.

The fourth reason why the claims could be considered as misleading is that they contradict recent studies. A systematic review by Roshanzamir and Safavi ^[12]^ identified 27 relevant studies, of which only four were in vivo human studies. They concluded that ‘more and high-quality randomized controlled trials need to be conducted on D-Asp supplementation regarding their ability to increase endogenous levels of testosterone and elucidating the mechanisms of its action in human research’.

The fifth reason for misleading the consumer is how the advertiser used bar graphs on their website to illustrate the results of the Topo et al.^[7]^ study. The bar graphs only show the upper half of the results and exaggerate the difference between the experimental and placebo groups. The advertiser starts the baseline at 3.5 for LH and 4 for testosterone instead of 0 (i.e. at the bottom of the graph). Only showing the upper parts of a bar graph accentuates an otherwise small difference. The advertiser did not mention the decline in the participant’s total testosterone three days after the suspension of D-aspartic acid (the data in the far right column, Table 1), and did not indicate this decline in the bar graph.

## Conclusion

When confronted with claims such as ‘100% clinically validated’ and ‘clinically researched’ the consumer could infer that the product has been subjected to relevant, unbiased and independent clinical trials, that it is safe and effective, and that there is sufficient scientific evidence to support these claims. From the manufacturer’s supplied information, it is clear that no such trials ever took place. The textual claims and the partial histogram misrepresent and exaggerate the scientific results, potentially misleading consumers. There are limited in vivo human studies on the effect of exogenous D-aspartic acid on testosterone blood serum.^[12]^ The evidence of the small and short duration studies is insufficient to conclude that D-aspartic acid leads to a substantial and clinically significant increase in testosterone levels. Problems related to the supplement industry are not only limited to regulatory matters, adulteration, contamination and unsubstantiated claims, but also include deception and potentially misleading advertising.

## Figures and Tables

**Table 1:** Effects of D-aspartate (D-Asp) on luteinising hormone (LH) and testosterone release in human serum (Topo et al.)^[7]^

	Basal levels treatment	After six days of D-Asp treatment	After 12 days of D-Asp	Three days after the suspension of D-Asp
**LH (mIU/ml serum)**				
Subjects treated with Na-D-aspartate	4.2 ± 0.5	4.5 ± 0.6	5.6 ± 0.9*	4.8 ± 0.8
Subjects treated with placebo	4.2 ± 0.4	4.3 ± 0.7	4.2 ± 0.6	4.1 ± 0.5
**Testosterone (ng/ml serum)**				
Subjects treated with Na-D-aspartate	4.5 ± 0.6	5.2 ± 0.7	6.4 ± 0.8*	5.8 ± 0.6*
Subjects treated with placebo	4.6 ± 0.5	4.5 ± 0.7	4.7 ± 0.7	4.6 ± 0.7

* Values are statistically significant for p < 0.001^[7]^
